# Tuberculosis of the Elbow Joint in an Indian Boy: A Rare Entity With a Diagnostic Challenge

**DOI:** 10.7759/cureus.58184

**Published:** 2024-04-13

**Authors:** Sankalp Yadav

**Affiliations:** 1 Medicine, Shri Madan Lal Khurana Chest Clinic, New Delhi, IND

**Keywords:** cartridge-based nucleic acid amplification test (cbnaat), diagnostic challange, mycobacterium tuberculosis (mtb), tuberculosis, elbow joint

## Abstract

Tuberculosis of the bones and joints is an infrequently reported entity. Isolated involvement of the elbow joint is exceedingly rare, even in endemic countries. The diagnosis is an arduous task, especially if it presents in younger age groups. Herein, a case of tuberculosis of the right elbow joint in a seven-year-old Indian child is presented. The diagnosis was challenging due to the vague clinical features and rarity of the disease, but he was diagnosed after a detailed clinical examination along with a radiometric assessment. He was initiated on the appropriate chemotherapy.

## Introduction

Tuberculosis remains a significant global issue, with about one-fourth of the world's population affected by *Mycobacterium tuberculosis* [1]. It stands as one of the most lethal infectious diseases, causing around 1.3 million deaths in 2022 alone [2]. Recent statistics from the World Health Organization for 2021 reveal that in India, the incidence and prevalence of tuberculosis are 188 and 312 per one lakh (0.1 million) population, respectively [3]. These figures underscore the ongoing challenge of tuberculosis control and the urgent need for effective interventions.

Osteoarticular tuberculosis often goes undiagnosed until significant bone damage has occurred, as its presentation varies. Around 20% of mycobacterial infections in children manifest as extrapulmonary tuberculosis, with bone or joint tuberculosis accounting for 5% of these cases [4]. The initial symptoms of extrapulmonary tuberculosis resemble other diseases, earning tuberculosis the moniker "a great imitator" [5].

About 30%-40% of extrapulmonary tuberculosis cases, and 1%-3% of all tuberculosis cases are attributed to musculoskeletal tuberculosis. The hip, knee, and other joints are the most frequently afflicted, followed by the spine [6]. Elbow joint tuberculosis is an uncommon condition, with an estimated 1%-5% of all musculoskeletal tuberculosis cases affecting the elbow joint [7]. Herein, a rare report of tuberculosis of the right elbow joint in an Indian boy is presented. The situation was challenging due to vague symptoms and the clinical course.

## Case presentation

A seven-year-old nondiabetic Indian boy was brought to the outpatient department by his parents with complaints of pain, reduced range of movement, and swelling over his right elbow for three months after a fall about eight months ago. The swelling was insidious in onset and tender to the touch. His parents consulted local clinicians for the last two months, but except for some temporary relief with unknown drugs, there was no substantial improvement.

There was no history of constitutional features of tuberculosis or a history of disease in the family. He was a student belonging to a middle-income family. There was no history of staying at crowded places like night shelters, juvenile homes, or refugee camps.

A general examination was suggestive of a hemodynamically stable boy. Local examination of the right elbow joint was suggestive of a swollen right elbow joint, tender to the touch with a range of motion for the elbow of 0° to 20°, painful within that range, and severely painful thereafter. The arc of supination to pronation was complete, albeit unsettling. Joint crepitus accompanied the movements. The distal neurovascular state remained unaltered. However, there were no discharging sinuses or raised local temperatures. The left elbow joint and the rest of his skeletal examination were unremarkable. There was no clubbing, cyanosis, koilonychia, epitrochlear, supratrochlear, or even axillary lymphadenopathy.

**Figure 1:**
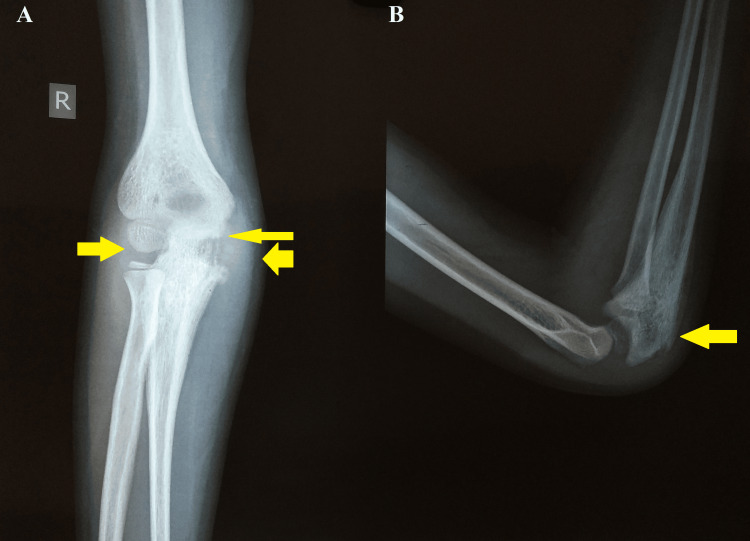
Anteroposterior and lateral radiographs of the right elbow joint radiographs showing erosion of the distal cortex of the humerus, olecranon process of the ulna, and radial bone head with swelling of the soft tissues around the right elbow joint A: Anteroposterior view (arrows showing erosion of the distal cortex of the humerus, olecranon process of the ulna, and radial bone head with swelling of the soft tissues around the right elbow joint). B: Lateral view

A plain radiograph of the right elbow joint demonstrated erosion of the distal cortex of the humerus, olecranon process of the ulna, and radial bone head with swelling of the soft tissues around the right elbow joint (Figure 1).

**Figure 2:**
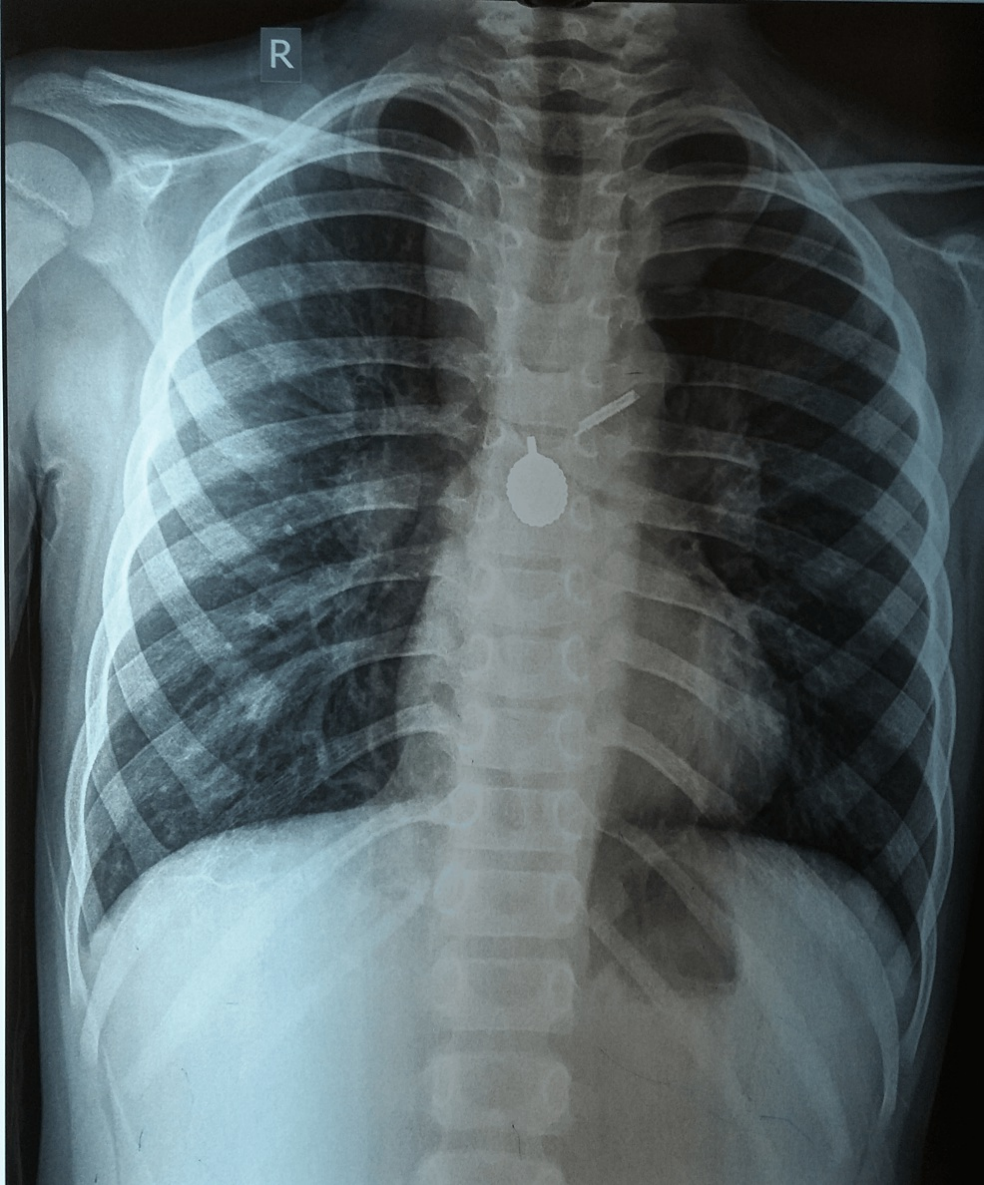
An unremarkable plain radiograph of the chest

A blood panel was suggestive of a minimally raised erythrocyte sedimentation rate of 38 mm/hour and C-reactive protein of 10 mg/L, but his HIV (I and II), rheumatoid factor, and hepatitis (A, B, and C) were negative. His Mantoux test was remarkable, with an induration of 20 x 15 mm. A chest radiograph was normal (Figure 2).

**Figure 3:**
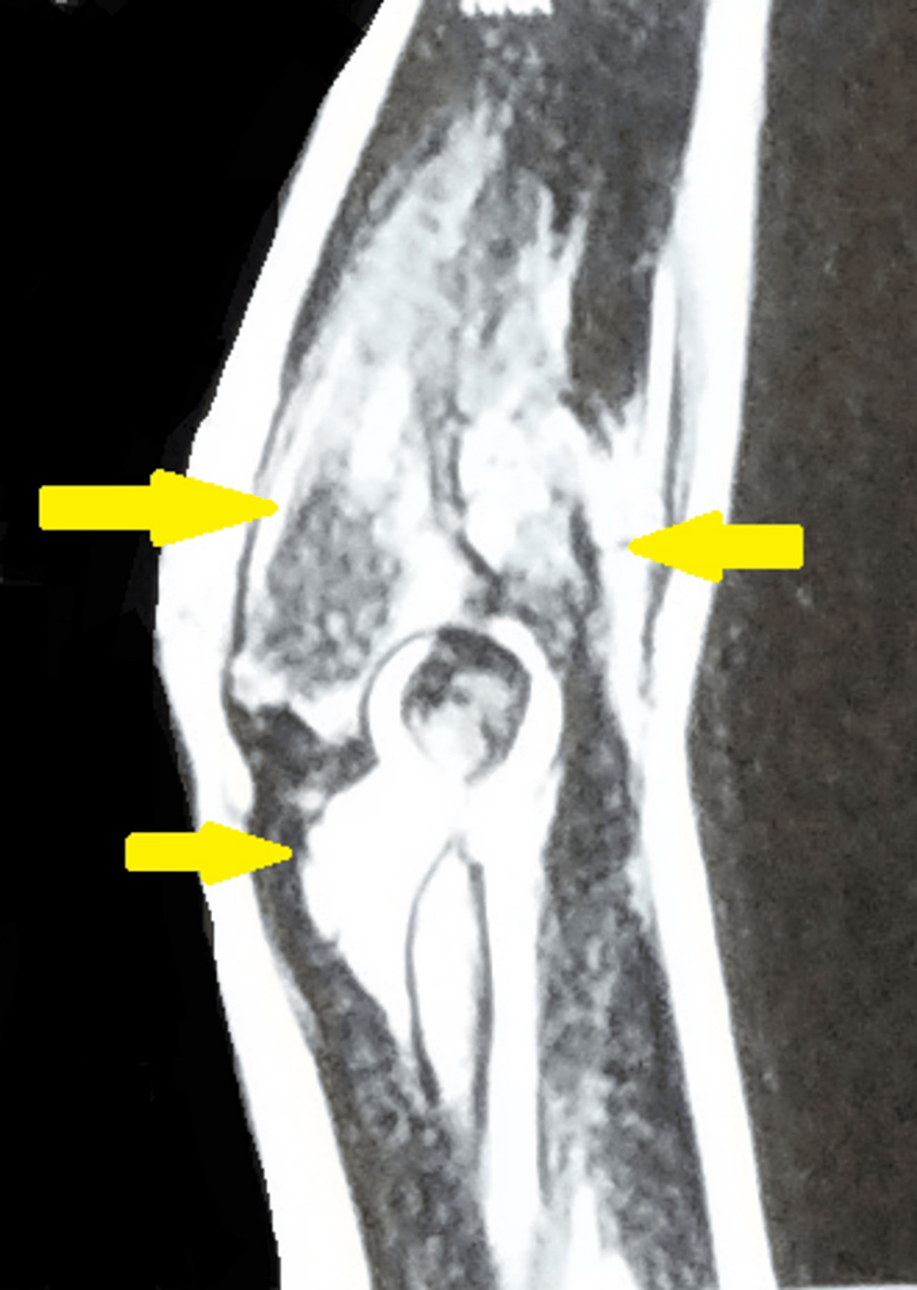
MRI of the right elbow was suggestive of altered signal intensity in the medial and lateral humeral condyle and epicondyle, the olecranon process of the ulna, trochlea, and radial head, and the opposing surfaces of the proximal radioulnar joint with partial loss of definition and widening of elbow joint space MRI: Magnetic resonance imaging

A magnetic resonance imaging of the right elbow was suggestive of altered signal intensity in the medial and lateral humeral condyle and epicondyle, the olecranon process of the ulna, trochlea, and radial head, and the opposing surfaces of the proximal radioulnar joint with partial loss of definition and widening of elbow joint space. Multiple loculated subcutaneous and intermuscular plane collections communicating with the joint space were seen in the medial aspect of the distal humerus, tracking posteriorly insinuating between the triceps and epicondylar attachment with edema and loss of definition of the triceps. This collection was noted anterior to the lateral epicondyle and radial head with edema in the brachialis and supinator, and a smaller one posterior to the lateral epicondyle. Associated effusion was seen distending the joint capsule with diffuse hyperintensity on the images and blurring of myofascial planes with associated subcutaneous edema (Figure 3).

A joint fluid was aspirated under sterile conditions, and it was sent for microscopy, a cartridge-based nucleic acid amplification test, and culture from an orthopedic minor operation theater. The results were remarkable for the very low detection of *Mycobacterium tuberculosis* with no rifampicin resistance on a cartridge-based nucleic acid amplification test. All the other tests were negative. Hence, a defined diagnosis of tuberculosis of the right elbow joint was made, and antituberculous chemotherapy was commenced in fixed-dose pediatric combinations per the national guidelines (intensive phase for two months with rifampicin, isoniazid, pyrazinamide, and ethambutol and 10 months of a continuation phase with rifampicin, ethambutol, and isoniazid). He was transferred to his village at his request with advice for treatment adherence and regular follow-ups in the orthopedics and infectious diseases outpatient department. His final outcome was marked as cured at 12 months of treatment in the national tuberculosis data portal.

## Discussion

Tuberculosis of the upper limb is seldom reported and is an infrequently encountered condition in outpatient departments [8]. Rarely are reports of elbow joint involvement found in the literature [6]. Elbow tuberculosis typically occurs as a result of hematogenous dissemination from a primary infection elsewhere in the body, primarily affecting children, which could be attributed to increased vascularity of the bones [6,9]. Moreover, the revival of a dormant focus following the emergence of a latent tuberculosis infection could also result in the disease [9]. Additionally, around 50% of cases with musculoskeletal tuberculosis may present a focus of disease in the lungs evident on chest radiographs, but the same was not seen in the present case [6].

Due to the lack of distinct systemic and pulmonary symptoms, elbow joint tuberculosis frequently manifests itself with a delay [10]. Studies show varying typical delays between the beginning of symptoms and diagnosis, ranging from one week to eight years, with a mean lag of 13 weeks [6].

Conditions like fungal infections, brucellosis, sarcoidosis, and leprosy share clinical and radiological features with osteoarticular tuberculosis, complicating diagnosis. Factors such as diabetes, immune deficiencies, and chronic diseases further obscure the diagnosis. In pediatric cases, delayed diagnosis is common due to a lack of awareness, nonspecific symptoms, and inconclusive imaging [11]. Constitutional symptoms may be absent in up to 72% of cases, adding to the diagnostic challenge [4].

Elbow tuberculosis is characterized by swelling, pain, and a progressive reduction in joint range of movement. Cold abscesses may serve as a crucial indicator of tuberculosis [6].

Diagnosis is mainly based on clinical assessment and radiometric investigations. Advanced radiometric investigations like computed tomography and magnetic resonance imaging (with a positive predictive value of nearly 96% in extrapulmonary tuberculosis) could help in establishing a definite diagnosis [4].

The cartridge-based nucleic acid amplification test, or GeneXpert-MTB (Cepheid, Sunnyvale, California), is a very sensitive and specific test (93%-95%) that can be used to help detect antimycobacterial resistance in addition to identifying *Mycobacterium tuberculosis* in samples. The gold standard is the detection of *Mycobacterium tuberculosis* and/or caseous granulomas from biopsy specimens by either Ziehl-Neelsen staining or fluorescent auramine staining [4]. However, because the illness is paucibacillary, the yield is poor [12].

Antituberculous medications are the mainstays of treatment [10]. They give outstanding results in children due to the high regenerating ability of the articular cartilage and epiphyses [13]. Surgical debridement for treating the condition is indicated only in advanced disease where fixed deformities at the joint are noted. Antitubercular chemotherapy usually lasts for a full year, though this can vary according to the severity and length of the illness [10,14]. It is critical to keep a close eye on patients' responses to treatment and any possible side effects from antitubercular medications [7].

Tuberculosis of the elbow is reported scarcely in the literature, and only a few cases with similar clinical presentations have been reported in pediatric patients [6]. Studies by Dix-Peek et al., Aggarwal et al., Agarwal et al., Agarwal et al., and Dhillon et al. documented such pediatric cases with tuberculosis of the elbow joint [15-19].

## Conclusions

A rare case of tuberculosis involving the elbow joint in a young boy is presented here. A high index of suspicion for skeletal tuberculosis, together with a thorough history taking and clinical and radiographic evaluation, can lead to an early diagnosis and effective therapy. This will maintain the joint's maximum function and assist in preventing osteoarticular degeneration.
